# Malignant Transformation Potentials of Human Umbilical Cord Mesenchymal Stem Cells Both Spontaneously and via 3-Methycholanthrene Induction

**DOI:** 10.1371/journal.pone.0081844

**Published:** 2013-12-10

**Authors:** Qiuling Tang, Qiurong Chen, Xiulan Lai, Sizheng Liu, Yezeng Chen, Zexin Zheng, Qingdong Xie, Martin Maldonado, Zhiwei Cai, Shan Qin, Guyu Ho, Lian Ma

**Affiliations:** 1 Transforming Medical Center, The Second Affiliated Hospital of Shantou University Medical College, Shantou, Guangdong, China; 2 Department of Pediatrics, The Second Affiliated Hospital of Shantou University Medical College, Shantou, Guangdong, China; 3 Research Center of Reproductive Medicine, Shantou University Medical College, Shantou, Guangdong, China; 4 Molecular Pathology Laboratory, Shantou University Medical College, Shantou, Guangdong, China; University of Newcastle, United Kingdom

## Abstract

Human umbilical cord mesenchymal stem cells (HUMSCs) are highly proliferative and can be induced to differentiate into advanced derivatives of all three germ layers. Thus, HUMSCs are considered to be a promising source for cell-targeted therapies and tissue engineering. However there are reports on spontaneous transformation of mesenchymal stem cells (MSCs) derived from human bone marrows. The capacity for HUMSCs to undergo malignant transform spontaneously or via induction by chemical carcinogens is presently unknown. Therefore, we isolated HUMSCs from 10 donors and assessed their transformation potential either spontaneously or by treating them with 3-methycholanthrene (3-MCA), a DNA-damaging carcinogen. The malignant transformation of HUMSCs in vitro was evaluated by morphological changes, proliferation rates, ability to enter cell senescence, the telomerase activity, chromosomal abnormality, and the ability to form tumors in vivo. Our studies showed that HUMSCs from all 10 donors ultimately entered senescence and did not undergo spontaneous malignant transformation. However, HUMSCs from two of the 10 donors treated with 3-MCA displayed an increased proliferation rate, failed to enter senescence, and exhibited an altered cell morphology. When these cells (tHUMSCs) were injected into immunodeficient mice, they gave rise to sarcoma-like or poorly differentiated tumors. Moreover, in contrast to HUMSCs, tHUMSCs showed a positive expression of human telomerase reverse transcriptase (hTERT) and did not exhibit a shortening of the relative telomere length during the long-term culture in vitro. Our studies demonstrate that HUMSCs are not susceptible to spontaneous malignant transformation. However, the malignant transformation could be induced by chemical carcinogen 3-MCA.

## Introduction

HUMSCs possess multipotent characteristics, have a relatively high proliferation rate, and can be induced to differentiate into advanced derivatives of all three germ layers, including osteoblasts, adipocytes, chondrocytes, myoblasts, islet cells, and neurons [1]–[7]. Studies have also shown that HUMSCs express low levels of HLA-ABC and do not express HLA-DR [8], [9], thus rendering them immunodeficient. Moreover, HUMSCs exhibit immunosuppressive activities in mixed lymphocyte assays [10]. The low-risk of host rejection, coupled with the large donor pool, rapid availability, and no ethical complications in use, makes HUMSCs a good cell source for use in regenerative medicine [11], [12].

However, recent controversies about the stability of human mesenchymal stem cells (hMSCs) from bone marrows [13]–[16] highlight the need to address the hMSC safety before clinical use. Roland et al. reported that spontaneous malignant transformation of bone marrow-derived hMSCs occurs in 45.8% (11 of 24) of cultures and concluded that spontaneous malignant transformation may represent a biohazard in long-term ex vivo expansion of hMSCs [17]. Similar phenomena of bone marrow-derived MSCs from both human and murine origins have also been reported in other studies [18]–[20]. But there is no knowledge thus far as to whether HUMSCs could undergo malignant transformation during long-term in vitro culture or could be induced to transform by carcinogens. Thus, we set out to investigate the risk of spontaneous malignant transformation of HUMSCs as well as the ability of 3-MCA, a DNA-damaging carcinogen [21], [22], to induce the HUMSC transformation.

## Materials and Methods

### 1. Isolation and culture of HUMSCs

All research involving human participants were reviewed and approved by the Medical Ethics Committee of Shantou University Medical College (Shantou, China). And all participants provided their written consent to participate in this study. Human umbilical cords were obtained from consenting patients delivering full-term infants by cesarean section at the Second Affiliated Hospital of Shantou University Medical College. HUMSCs were isolated from Wharton's jelly as described before [23]. Briefly, HUMSCs were isolated by culturing Wharton's jelly in the high glucose Dulbecco' modified essential media (H-DMEM; Gibco, USA) containing 15% fetal bovine serum (FBS; Gibco, USA), 100 u/ml penicillin and 100 mg/ml streptomycin. After 7 days of culture, cells reached 90% confluence and were transferred in DMEM with low glucose and 10% FBS. All clinical investigation have been conducted according to the principles expressed in the Declaration of Helsinki.

### 2. Treatment of HUMSCs with 3-MCA

3-MCA (Sigma) was dissolved in dimethyl sulfoxide (DMSO; Sigma) at a concentration of 1 g/ml, stored at 4°C in the dark. The third passage of HUMSCs (P3) was divided into two groups. One was treated with 5 ug/ml 3-MCA in culture medium (DMEM containing 20% FBS) for one week, after which the medium was changed to culture medium without 3-MCA twice per week. Another group was treated with 0.5% DMSO as the control. These two groups were monitored morphologically under an inverted microscope and the cell proliferation rate as assessed by the population doubling time (PDT) was calculated as described by Shaffer et al. [24]. PDT = (T×ln2)/ln (Nf/Ni), where T is cell culture time, Ni is the number of cells after 24 h of seeding and Nf is the number of cells after 4 days of seeding.

### 3. Cell proliferation assay

The cell proliferation assay was carried out as described by Stute [25] with minor modifications. Briefly, 1000 cells were seeded in quintuplicate in 96-well plates. MTT (3-(4,5-dimethylthiazol-2-yl)-2,5-diphenyl tetrazolium bromide, Beyotime Institute of Biotechnology) was dissolved in PBS at 5 mg/ml and filtered to sterilize. The stock MTT (10 ul per 100 ul medium) was added to wells, and plates were incubated at 37°C for 4 h. Thereafter, the supernatant was removed from the well (100 ul) and 150 ul DMSO was added to dissolve the dark blue crystals for 10mins before the plates were read on a microplate reader (Bio-Rad) at the wavelength of 490 nm. The OD readings were analyzed statistically using Analyze-it software from Microsoft Excel.

### 4. β-galactosidase (β-gal) staining

Increased endogenous β-gal activity at pH 6.0 was used to determine the state of cell senescence according to the β-gal assay protocol (Beyotime Institute of Biotechnology). The characteristic blue staining of cells with high β-gal activity was examined by light microscopy.

### 5. In vivo tumor formation

Immunodeficient Balb/C mice from the Animal Experiment Center of Guangzhou University of Chinese Medicine were used. All experiments were approved by Shantou University Medical College Animal Research Committee. The mice were injected subcutaneously with 10^6^ tHUMSCs. The control group was injected with equal numbers of HUMSCs. Each group consisted of three mice. The animals were monitored and weighed daily throughout the experiment.

### 6. Tumor tissue collection and analysis

As soon as the animals lost 10% body weight or exhibited subcutaneous tumor growth, they were sacrificed by cervical dislocation. Tumors were excised and embedded in paraffin, sectioned, and stained with hematoxylin-eosin (HE) before examination by microscopy.

### 7. Immunohistochemistry

Immunohistochemistry was carried out using the standard protocol. Retrieval method, buffer solutions, antibody detection, and concentration of the primary antibodies were varied according to manufacturers' instructions described for each primary antibody. The various dilutions of primary antibodies were as follows: vimentin (Vim,7 ug/ml; ZSGB-BIO), desmin (Des, 1∶500; ZSGB-BIO), cytokeratin (CK, 1∶400; ZSGB-BIO), epithelial membrane antigen (EMA, 1∶200; ZSGB-BIO), neurofilament (NF, 1∶200; ZSGB-BIO), Ki-67 (1∶500; ZSGB-BIO) and CD99 (1∶500; ZSGB-BIO). Immunodetection was performed using HistoMouse-SP Kit (AEC, Broad Spectrum). Finally, the slides were counterstained with hematoxylin for 1 min, dehydrated, and mounted with resinene. A Leica DMLB microscope was used to examine the staining and pictures were taken with the ColorView soft imaging system (Olympus).

### 8. Isolation and culture of tumor cells

Tumor tissues removed from the nude mice were washed in PBS containing 200 u/ml penicillin and 200 u/ml streptomycin. After cutting into 1 mm^2^ pieces, the tumor tissues were digested with trypsin for 30 min, stirred frequently, and followed by adding FBS to terminate the digestion. The cell suspension was immediately centrifuged, resuspended in DMEM containing 10% FBS, and cultured in 37°C, 5% CO_2_ incubator. The medium was changed after cells became adherent to culture flasks.

### 9. Karyotype analysis

Prior to harvest, cells in culture were incubated with colchicine at 0.4 ug/ml for 4 h, resuspended in 0.075 M KCl at 37°C for 20 min, and then fixed in methanol/acetic acid (3∶1) at room temperature for 20 min. After centrifugation, the supernatant was removed. Cells were then fixed again with methanol/acetic acid for another 20 min. The cell solution was dropwise added onto the slides. The Trypsin-Giemsa banding was carried out by Genetix GSL-120 automatic imaging system. And at least 10 metaphase cells were analyzed by the CytoVision Version 4.5.1 Build 5 software.

### 10. RNA extraction and RT-PCR analysis of hTERT

Total cellular RNA was extracted using Trizol reagent (Kakara, Japan). cDNA synthesis was performed with a cDNA kit according to the manufacturer's protocol (Kakara, Japan). The hTERT cDNA amplification used primers TERT-1784 (forward, CGGAAGAGTGTCTGGAGCAA) and TERT-1928 (reverse, GGATGAAGCGGAGTCTGGA) with an initial heating at 94°C for 90 s, followed by 35 cycles of 95°C for 25 s, 68°C for 50 s, and 72°C for 50 s. The PCR products were analyzed using 1.5% agarose gel electrophoresis and visualized via ethidium bromide staining.

### 11. Real-Time quantitative PCR (qPCR) analysis of the relative telomere length

Genomic DNA was isolated using Nucleic Acid Purification kit (DSBIO, China). The relative telomere length was determined by qPCR as described before [26], [27]. Briefly, the single copy gene 36B4 (S) was used as a reference to determine the relative copy numbers of telomere (T) in each sample. The ratio of T to S (the relative telomere length) is shown to be directly proportional to the telomere length [26], [27]. Standard curves for telomere and 36B4 qPCRs were generated by a serial 2-fold dilution of genomic DNA samples starting from 160 ng/ul to 5 ng/ul to produce optimal R^2^ and slope for suitable calculation of ΔΔCt of qPCR results.

Triplicate PCR reactions using 2 ul of each DNA dilution (60 ng) were carried out in a 20 ul volume using the SYBR Premix Ex Taq™ kit (Takara, Japan). The final concentrations of primers were: tel forward (CGGTTTGTTTGGGTTTGGGTTTGGGTTTGGGTTTGGGTT), 270 nM; tel reverse (GGCTTGCCTTACCCTTACCCTTACCCTTACCCTTACCCT), 900 nM; 36B4 forward (CAGCAAGTGGGAAGGTGTAATCC), 300 nM; 36B4 reverse (CCCATTCTATCATCAACGGGTACAA), 500 nM. These primer sequences have been described previously [26].

All qPCRs were performed on the Prism 7300 Sequence Detection System (Applied Biosystems, America). The reaction was activated at 95°C for 10 min, followed by 30 cycles of 95°C for 15 s, 54°C for 2 min in the case of the telomere amplification or followed by 40 cycles of 95°C for 15 s, 58°C for 1 min in the case of the 36B4 amplification. ABI's SDSS v1.2.3 software was used to generate the standard curve.

## Results

### 1. Phenotypic characterization of HUMSCs during long-term in vitro culture

**Figure 1:**
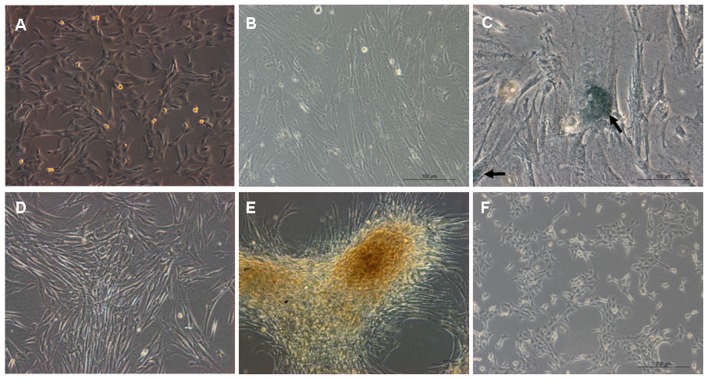
Phenotypic characterization of HUMSCs and tHUMSCs. HUMSCs during continuous in vitro culture maintained the typical spindle shape at P3 (A) and P15 (B). (×100) Senescence-associated β-gal staining (arrows) was positive at P15 (C) (×200). tHUMSCs appeared to grow in clusters at day 7 (D) and in layers at day 120 (E) after treating with 3-MCA. At a lower cell density, tHUMSCs appeared round during continuous expansion (F). (×100).

After 5–7 days of starting the HUMSC primary culture, spindle-shape cells started to migrate out from fragments of Wharton's jelly. Primary HUMSCs were not split until 10–14 days later when cells reached 80%–90% confluence. HUMSCs exhibited a typical fibroblast-like morphology (Fig. 1A). We showed before that these cells express cell-surface antigens associated with multipotent stem cells such as OCT4 CD29, CD44, and CD 59 [23].

**Figure 2:**
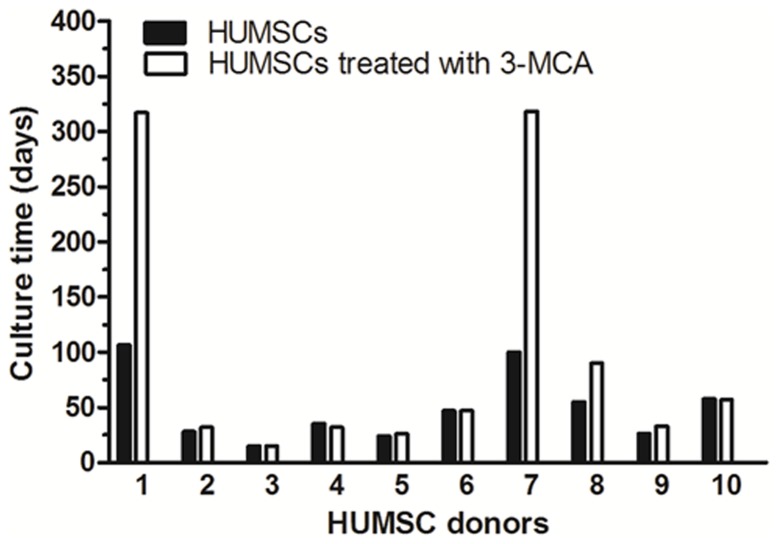
In vitro life span of donor HUMSCs. The life span of cells was defined as the number of culture days before observation of senescence. Donor HUMSCs were treated with or without 3-MCA. HUMSCs treated with 3-MCA from donors 1 and 7, while still growing, were terminated at 318 days to allow for data analysis.

Following an extended period culture of HUMSCs in vitro, we did not observe spontaneous malignant transformation of HUMSCs from all 10 donors. However, there was a wide variability between donor HUMSCs in their proliferative capacity and in vitro life span. Donors 2–6 and 8–10 entered senescence with a mean culture time of 36 days, with the shortest time of 15 days to the longest time of 58 days (Fig. 2). The cell senescence was assessed by β-gal staining. Senescence-associated β-gal activity, detectable at pH 6.0, permits the identification of senescent cells in culture and tissues [28]. As shown in (Fig. 1C), One donor HUMSCs at P15 showed positive staining for β-gal, while the same HUMSCs at P3 were negative for the β-gal activity (data not shown). The average cell PDT increased from 1.79±0.24 to 3.77±0.46 over a period of 4 days. Donors 1 and 7 showed a relatively late growth arrest, which entered cell senescence with a culture time of 107 days for donor 1 and 100 days for donor 7. However, their average cell PDT did not significantly differ from that of the other donors (an increase from 1.89±0.28 to 3.97±0.23). The mean culture time form all donor HUMSCs were 49.5 days before entering senescence and all donor cells maintained the typical spindle-shaped morphology at the senescence phase and progressively died. Using the criteria of positive β-gal activity, increased cell PDT, lack of apparent morphological changes, and eventual cell death, we concluded that HUMSCs did not undergo spontaneous transformation.

### 2. Malignant transformation of HUMSCs treated with 3-MCA

All 10 donor HUMSCs were also treated with 3-MCA to assess their transformation potential under the carcinogen. Approximately between 10 to 20 days after the 3-MCA treatment, HUMSCs started to display sick characteristics, where they showed weak cell refraction under light microscopy, exhibited cytoplasmic vacuoles and granules (data not shown). The “sick” phenotype was presumably due to the cytotoxicity of 3-MCA. Cells in the “sick” phase varied in length from 10 to 30 days. HUMSCs from eight of the 10 donors eventually died.

**Figure 3:**
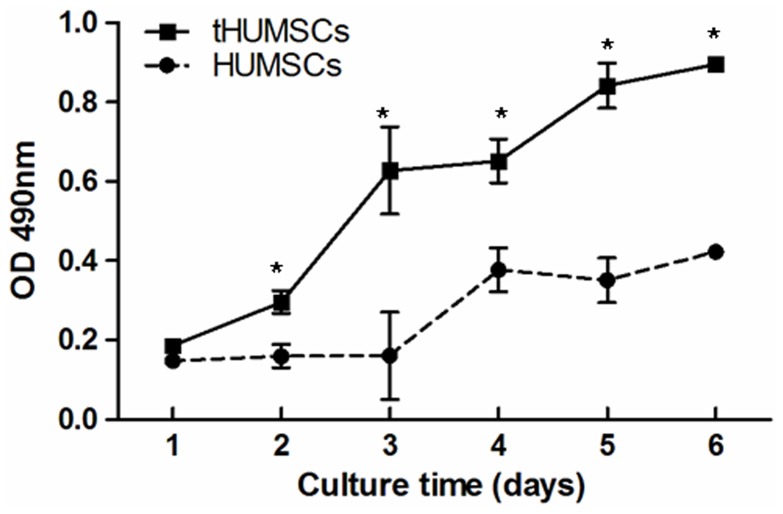
Cell proliferation analysis. HUMSCs and tHUMSCs were seeded in culture plates and the cell proliferation was assessed daily by MTT for 6 days. The proliferation rate was significantly increased in tHUMSCs compared with HUMSCs from day 2 to day 6. *P<0.01.

However, HUMSCs from two donors (1 and 7) survived the 3-MCA treatment and propagated over 300 days (Fig. 2). Cells from these two donors had similar growth characteristics and were named tHUMSCs. tHUMSCs showed a significantly increased proliferation rate, when measured by MTT assays, as compared to that of HUMSCs (Fig. 3). tHUMSCs also exhibited a decreased PDT from 3.89±0.33 to 1.77±0.34 over a 4-day period. In addition, they tend to grow in clusters and layers (Fig. 1D, E) and after growing in culture for 120 days, they had lost the fibroblast-like shape and become round (Fig. 1F).

### 3. Tumorigenicity

**Figure 4:**
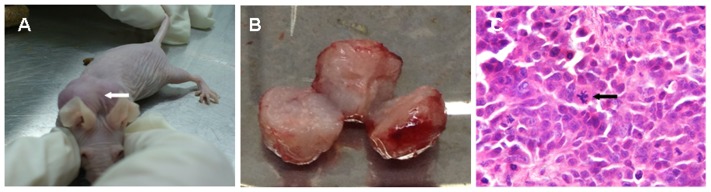
Tumor growth in vivo. (A) tHUMSCs formed tumors in the subcutaneous layer of the immunodeficient mice. (B) The tumor tissue exhibited fish-like texture. (C) HE staining showed round atypical cells, enlarged nuclei, decreased karyoplasm, and elevated mitotic index (arrow). (×200).

To evaluate tumorigenic ability, tHUMSCs were injected subcutaneously into BALB/c-nu/nu mice. One of the three animals injected with the cells visibly formed tumors after 30 days in the position of injection (Fig. 4A). Control animals injected with HUMSCs at P4 and P10 were sacrificed 3 months post-injection and no signs of tumor development were detected.

**Figure 5:**
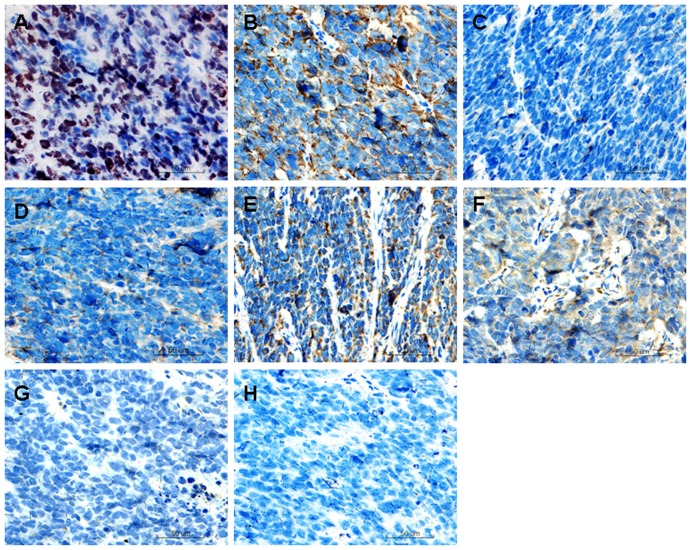
Immunohistochemical analysis of the tumor tissue. The tumor tissue was positive for cell proliferative marker Ki67 (brown nuclear staining) (A) mesenchymal tissue markers Vim (B), CK (C), and CD99 (D), neural tissue markers NF (E) and NES (F) (brown cytoplasmic staining), but it was negative for epithelial markers EMA (G) and Des (H). (×200).

The tumor tissues were excised and examined grossly and histologically. On gross examination, the tissue exhibited fish-like texture (Fig. 4B). HE staining identified malignant characteristics such as atypical round cells, enlarged nuclei, decreased karyoplasm, elevated mitotic index (Fig. 4C). We also examined, by immunohistochemistry, the expression of a series of mesenchymal and ectodermal tissue markers to evaluate the nature of the tumors. As shown in Fig. 5, tumor cells expressed markers of mesenchymal tissue Vim, CK, and CD99. In addition, they were positive for neural tissue markers NF and NES. However, there were negative for epithelial markers EMA and Des. We also stained cells with Ki67, a cell proliferation marker, and observed that the Ki67 index (a ratio of Ki67 positive cells to Ki67 negative cells) was more than 50%, indicating a highly proliferative nature of the cells. Taken together, these results indicate that tHUMSCs had given rise to sarcoma-like or poorly differentiated tumors.

### 4. Karyotype analysis

**Figure 6:**
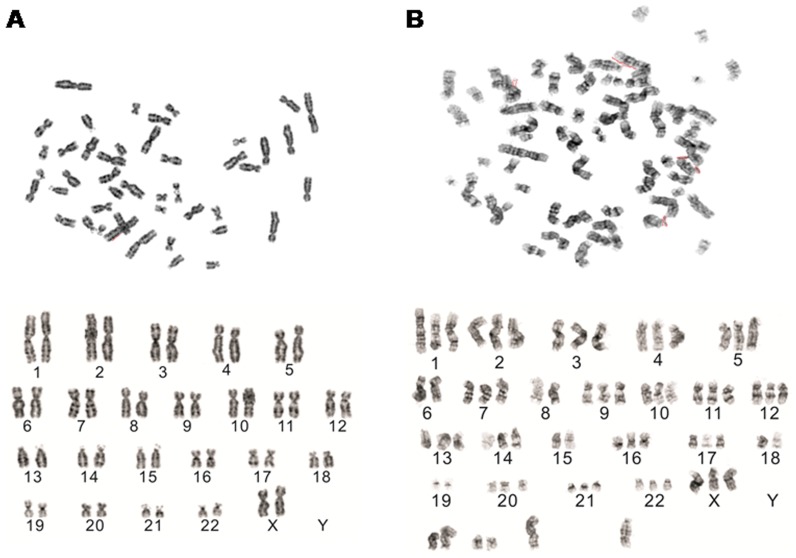
Karyotype analysis. (A) HUMSCs exhibited a normal Karyotype (46, XX). (B) A representative karyotype of tHUMSCs exhibited triploid and hypotriploid with 69 chromosomes. (×1000).

Karyotype analysis was performed for HUMSCs as well as for tHUMSCs. HUMSCs exhibited a karyotype of 46 chromosomes, which could be arranged into the normal 23 pairs (46, XX) (Fig. 6A). However, of the 10 tHUMSCs metaphases analyzed, chromosomes exhibited triploid and hypotriploid with chromosome numbers ranging form 61–69. In addition, some of the chromosomes did not show either a clear banding system or clear centromere location, thus could not be assigned to any one of the 23 pairs of human chromosomes. A representative karyotype of tHUMSCs was shown in Fig. 6B. Because the karyotye of tHUMSCs was different from that of mouse which has 40 acrocentric chromosomes, the result also confirmed the human cell origin of the tumor.

### 5. Analysis of telomere length

The relative telomere length was determined by qPCR as described by Cawthon et al. [26]. The slopes of the standard curves generated for the single copy gene 36B4 and telomere gene were −3.422 and −3.147, respectively. The regression coefficient (R^2^) was 0.97 for both qPCR reactions. Thus, the parameters were suitable for the linear fit analysis of the relative telomere length.

**Figure 7:**
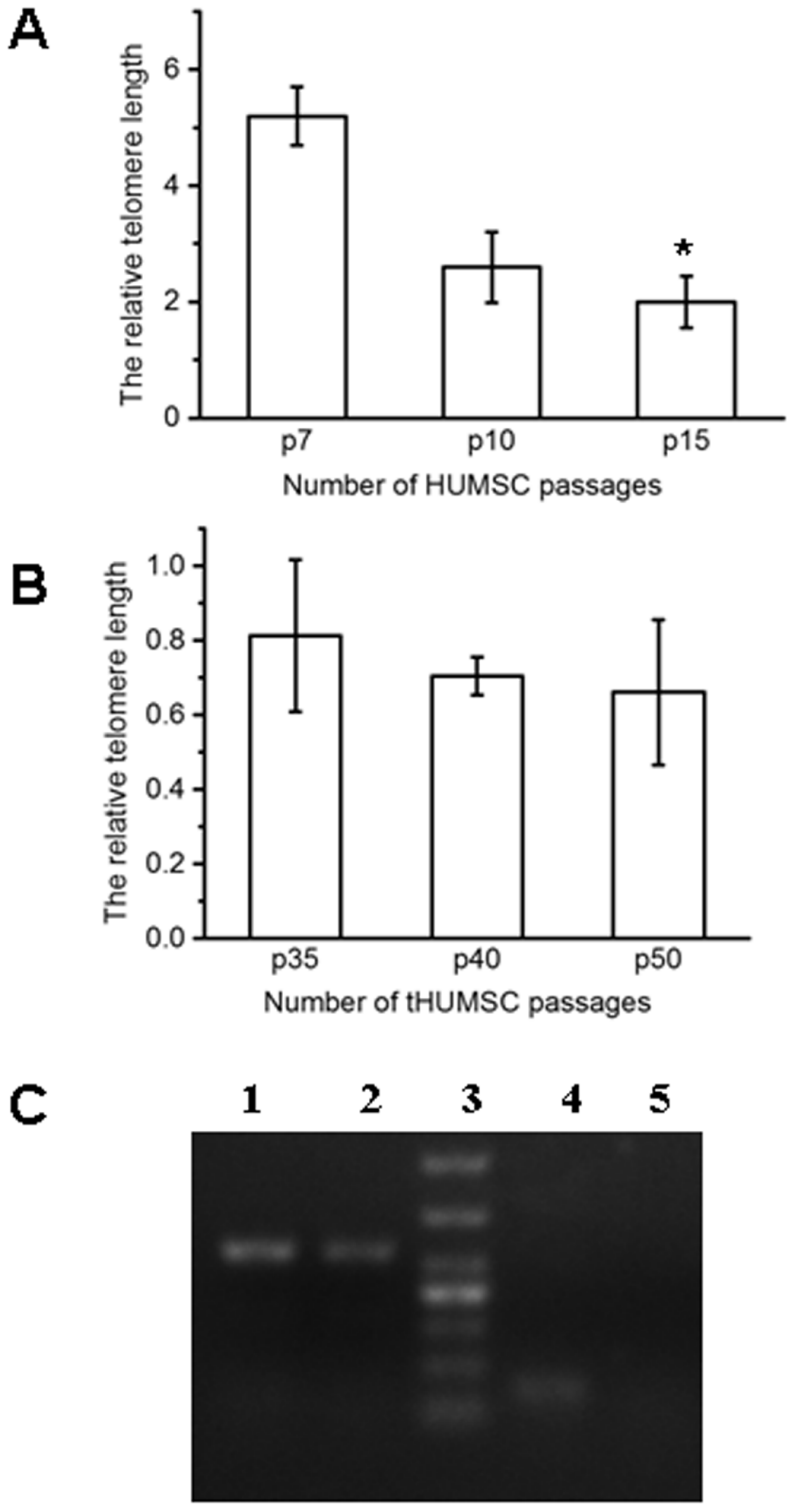
Analysis of telomerase activity. (A) The relative telomere length of HUMSCs was measure by qPCR, which was decreased as the cell passage number increased. *P<0.05 (B) There was no statistical difference in the relative telomere length of tHUMSCs among passages as analyzed by qPCR. (C) RT-PCR revealed the expression of hTERT in tHUMSCs at P50 (lane 4), while HUMSCs showed no detectable expression of hTERT at P3 (lane 5). The transcripts of GAPDH (glyceraldehyde-3-phosphate dehydrogenase) from tHUMSCs (lane 2) and HUMSCs (lane 1) were used as positive controls.

The relative telomere length from one of the representative donor HUMSCs at P7, P10, and P15 were 5.197±0.506, 2.596±0.629, and 1.997±0.442, respectively (Fig. 7A). There was a statistically significant difference between P7 and P15, demonstrating a gradual shortening of the telomere as HUMSCs grew in vitro. In contrast, the relative telomere length for tHUMSCs at P35, P40, and P50 were 0.812±0.204, 0.705±0.050, and 0.661±0.194, respectively (Fig. 7B), which was not statistically different between the passages. Noticeably, the relative telomere length was significantly shorter in tHUMSCs than that of HUMSCs (P<0.05).

### 6. hTERT expression

A high hTERT expression is required to maintain the telomerase activity for continuous cell proliferation [29]–[31]. We therefore investigated the expression of hTERT by RT-PCR. The expression of hTERT was not detected in HUMSCs at the early passage P3 (Fig. 7C). HUMSCs at subsequent passages P4, P6, and P10 were also examined and no hTERT expression was detected (data not shown). However, tHUMSCs expressed hTERT even at the late passage P50 (Fig. 7C). The expression at earlier passage P35 was also positive (data not shown).

## Discussion

HUMSCs are primitive stromal cells that have been shown to differentiate into cell types of all three germ layers [32]. In addition, HUMSCs exhibit low immunogenicity [8], [9] as well as immunosuppressive activities [10]. Thus, HUMSCs are considered as a promising cell source for use in regenerative medicine. To examine the biosafety of HUMSCs, we isolated HUMSCs from 10 donors and cultured them in vitro to assess their malignant transformation potentials spontaneously or via the 3-MCA induction.

Our results showed that HUMSCs from all 10 donors propagated in culture continuously for up to 15–20 passages with mean duration of 49.5 days before entering senescence and ultimately died. None of these donor cells underwent spontaneous malignant transformation. However, when HUMSCs were treated with 3-MCA, HUMSCs from two of the 10 donors which had survived the 3-MCA treatment underwent malignant transformation and were capable of growing in culture for the entire study duration of over 300 days.

The 10 donors exhibited different proliferation abilities (Fig. 2). We assume that the difference between donors might be due to individual differences. However, we also found that HUMSCs from donor 1 and 7, which had greater proliferation ability, had a higher potential to be transformed by the chemical carcinogen. The observation appeared to be in line with the notion that there is a greater chance for mutations to accumulate in long-term growing cells than short-term growing ones [43].

All donor HUMSCs showed a progressive decline in cell proliferative rate with an increase in the cell PDT. This is in agreement with the previous study where a decreased cell growth rate was observed in HUMSCs and bone marrow-derived MSCs as the cells were expanded in culture [33]. We further showed that HUMSCs entered senescence at variable culture times between 15 to 58 days (Fig. 1B, C) as evaluated by senescence-associated β-gal assays. HUMSCs from two donors (1 and 7), though being in the senescence phase for 50–70 days, could not be propagated further and progressively died (with a survival time of 100 and 107 days). Moreover, HUMSCs from all donors did not express hTERT as assessed by RT-PCR (Fig. 7C), an enzyme that reflects the telomerase activity in cells [34]. HUMSCs also exhibited progressive telomere shortening during cell proliferation (Fig. 7A). These data suggest that HUMSCs are not susceptible to malignant transformation after extensive in vitro expansion. Our finding is similar to that observed from hMSCs derived from chorionic villi and amniotic fluid [35], however, it contrasts from the observation of MSCs derived from bone marrows, where high rates of spontaneous malignant transformation were observed during in vitro cell expansion [17]–[18], [20].

In this study, we also investigated the transformation potential of HUMSCs induced by chemical carcinogen 3-MCA. We chose 3-MCA as the carcinogen for the following reasons: 1) 3-MCA is one of the most potent polycyclic aromatic hydrocarbon (PAH) carcinogens [21]. Metabolism of 3-MCA by cytochrome P450 (P450) enzymes leads to the formation of chemically reactive intermediates that can bind covalently to DNA, a critical step in the initiation of carcinogenesis [22]. It has been widely used as a model in chemical carcinogenesis studies [41]; 2) A previous study on the effect of 3-MCA or X-ray radiation on the occurrence of the mammary carcinoma in rats shows that both 3-MCA and X-ray irradiation are potent carcinogens, and 3-MCA is even more so [42]. Liu et al [36] reported that murine bone marrow-derived MSCs treated with 3-MCA undergo malignant transformation and the transformed cells form various types of tumors, such as epithelial tumors, neural tumors, and muscular tumors. In our study, HUMSCs from two donors which had survived the 3-MCA treatment were capable of in vitro expansion for over 300 days (Fig. 2). Morphologically, HUMSCs maintained the typical fibroblast-like shape throughout the entire culture period. In contrast, tHUMSCs grew in layers and clusters, assumed round cell shape (Fig. 1D, E, F), and displayed an increased proliferation rate (Fig. 3). tHUMSCs, when inoculated in the immunodeficient mice, resulted in tumor formation at the site of injection (Fig. 4A). When evaluated by immunohistochemistry, the tumor tissue expressed mesenchymal cell makers Vim, CK, and CD99, neural cell markers NF and NES, but was negative for epithelial markers EMA and Des (Fig 5). Thus, it appeared that tHUMSCs gave rise to sarcoma-like or poorly differentiated tumors. The result is in line with the primitive nature of HUMSCs.

Our study further showed that tHUMSCs were positive for hTERT expression, indicating a high cellular telomerase activity [29]–[31]. Consequently, the relative telomere length of tHUMSCs remained unchanged throughout the extended culture time (Fig. 7B). This phenomenon is in agreement with the finding that tumor cells are capable of maintaining the telomere length due to the high telomerase activity in cells [15], [16], [18]. Noticeably, we observed that the relative telomere length of tHUMSCs was shorter than that of HUMSCs (Fig. 7A, B). It is commonly accepted that the telomere length correlates with the cell proliferative capability in non-immortalized cells [37], [38]. However, in tumor cells it has been observed that chromosome terminal restriction fragments (TRFs), a measure of telomere length, can be drastically reduced to 2–4 kb [39], which is in shape contrast to that of highly proliferative germline or fetal cells where the TRFs are over 20 kb [40]. Thus, it is possible that tHUMSCs, which had assumed tumor biological characteristics, have lost the telomere feature of HUMSCs.

In conclusion, our data showed that HUMSCs propagating in continuous culture ultimately entered senescence and were not susceptible to spontaneous malignant transformation. The data suggest the biosafety of expanding HUMSCs in vitro for use in regenerative medicine. However, when HUMSCs were treated with the DNA damaging carcinogen 3-MCA, they did undergo malignant transformation and gave rise to poorly differentiated tumors in vivo.
